# Low nucleotide diversity of the *Plasmodium falciparum* AP2-EXP2 gene among clinical samples from Ghana

**DOI:** 10.1186/s13071-024-06545-6

**Published:** 2024-11-05

**Authors:** Elvis Quansah, Ji Zhao, Kenneth Kofi Eduful, Enock Kofi Amoako, Lucas Amenga-Etego, Faustina Halm-Lai, Qingli Luo, Jilong Shen, Chao Zhang, Li Yu

**Affiliations:** 1 Department of Microbiology and Parasitology, Anhui Province Key Laboratory of Zoonoses, School of Basic Medical Sciences, Anhui Medical University, Hefei, 230032 People’s Republic of China; 2 Akenten Appiah Menka University of Skills Training and Entrepreneurial Development, Asante Mampong, Ghana; 3 Department of Medical Laboratory, Health Service Directorate, Cape Coast Technical University, Cape Coast, Ghana; 4 West African Centre for Cell Biology of Infectious Pathogens (WACCBIP), Accra, Ghana; 5 Department of Biochemistry, Cell and Molecular Biology, West African Centre for Cell Biology of Infectious Pathogens (WACCBIP), University of Ghana, Legon, Accra, Ghana; 6 Department of Microbiology and Immunology, School of Medical Sciences, University of Cape Coast, Cape Coast, Ghana

**Keywords:** Malaria, *Plasmodium falciparum*, Transcriptional factor *AP2-EXP2*, Nucleotide variation

## Abstract

**Background:**

*Pf*AP2-EXP2 is located within chromosome 6 of *Plasmodium falciparum* recently identified to be undergoing an extensive selective sweep in West African isolates. The gene encoding this transcription factor, *Pf*AP2-EXP2, is essential and thus likely subject to purifying selection that limits variants in the parasite population despite its genomic location.

**Methods:**

72 *Plasmodium falciparum* field samples and 801 clinical sequences from the Pf6 MalariaGEN dataset of Ghanaian origin, were integrated and analysed.

**Results:**

A total of 14 single nucleotide variants of which 5 were missense variants, were identified after quality checks and filtering. Except for one, all identified variants were rare among the clinical samples obtained in this study (Minor allelic frequency < 0.01). Further results revealed a considerably low dN/dS value (0.208) suggesting the presence of purifying selection. Further, all the mutant amino acids were wildtype residues in *AP2-EXP2* orthologous proteins—tentatively suggesting a genus-level conservation of amino acid residues. Computational analysis and predictions corroborated these findings.

**Conclusions:**

Despite the recent extensive selective sweep within chromosome 6 of West African isolates, *Pf*AP2-EXP2 of Ghanaian origin exhibits low nucleotide diversity and very low dN/dS consistent with purifying selection acting to maintain the function of an essential gene. The conservation of AP2-EXP2 is an important factor that makes it a potential drug target.

**Graphical Abstract:**

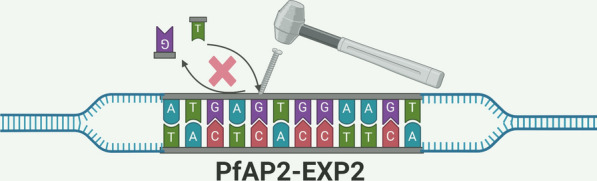

**Supplementary Information:**

The online version contains supplementary material available at 10.1186/s13071-024-06545-6.

## Background

Analysis of genomic variation in many different *Plasmodium* species provides opportunities to understand the basis of complex traits, including transcription control and antimalarial resistance. Tight transcriptional control by transcriptional factors in *Plasmodium* ensures effective morphological transitions and survival within mammalian and vertebrate hosts [1, 2]. Recently, transcription factors have emerged as possible antimalarial drug targets [3, 4]. Indeed the development of future antimalarial drugs targeting transcriptional factors would hugely rely on a comprehensive understanding of the biology and the dynamics of structural changes exhibited by these factors within clinical isolates.

The key concept of tight transcriptional control by a major class of transcription factors, ApiAP2, in Apicomplexans like *Plasmodium* was established about two decades ago [5]. The pioneering study established that this group of putative transcription factors has a DNA binding domain similar to the Apetala2/ERF (ethylene response factor) found in many plant transcription factors [5, 6]. Fast forward, an increasing number of studies have characterised many members of *Plasmodium falciparum* ApiAP2 (*Pf*ApiAP2) to control the transcription of a wide array of genes critically needed for key cellular processes throughout the parasites’ life cycle [7–10].

To maintain precise transcriptional control, genes encoding transcription factors are subjected to purifying selection that removes deleterious variants from the population. Nucleotide variation in the coding sequences results in the production of mutant proteins compatible with, or deleterious to, its protein stability [11]. The occurrence of nucleotide change in transcription factors could lead to misregulation of regulons. Non-deleterious changes, nonetheless, may still influence protein function via different mechanisms, such as perturbing active site residues of key binding protein/DNA binding sites [12, 13]. Such effects can be captured through the direct exploration of associations between genetic variants and parasite factors or between genetic variants and host phenotypes. Although the conservation of the structural integrity of transcription factors is beneficial for the parasites, it however presents a good feature against which drugs can be targeted.

Elucidating the effect of genetic variations has been a key method for unravelling the functions of ApiAP2 member proteins in laboratory clones of *Plasmodium* parasites [14, 15]. PFD7_0611200 also known as *Pf*AP2-EXP2 is a member of the ApiAP2 protein family. It is composed of 278 amino acids encoded by three exons and has a single DNA-binding domain. *Pf*AP2-EXP2 is located at genomic position 467227-469157 on chromosome 6. A mounting of recent evidence from *Plasmodium falciparum* (*P. falciparum*) originating from endemic West African sub-regions (Senegal and The Gambia) suggests that chromosome 6 is currently experiencing an extensive selective sweep with exceptionally long-range haplotypes [16, 17]. Interestingly, a sister ApiAP2 member, PF3D7_0613800, proximal to AP2-EXP2 on chromosome 6 (genomic position of 566139-578993) has been inferred to show a signal of positive selection in *P. falciparum* isolates originating from Togo [18]. However, of the many genes on chromosome 6 identified to be undergoing selective sweep, AP2-EXP2 was not included. This, perhaps, reflects the high recombination rate of *P. falciparum* [19] which limits the extent of selective sweeps such that purifying selection can still operate on essential genes without hitchhiking of deleterious mutations [20].

AP2-EXP2 is a key transcriptional factor putatively involved in controlling cellular remodelling and RBC invasion-related genes via repression of its regulons [21, 22]. A conditional knockdown of this gene resulted in growth defect indicating that this gene is likely essential. In the same study, the *Pf*AP2-EXP2 was refractory to gene knockout supporting the claim that this gene is essential. The *P. berghei* AP2-EXP2 ortholog (PBANKA_0109500) could not be knocked out in the Modrzynska et al. study [7]. In light of this, in clinical *P. falciparum* samples, it is expected that the essentiality of *Pf*AP2-EXP2 would subject it to purifying selection which removes deleterious variants. Therefore most variants are neutral and thus not expected to show phenotypic associations with host factors in clinical samples. Here we sought to explore the nucleotide diversity and the presence of selection in *Pf*AP2-EXP2 in Ghanaian clinical isolates. Further, we explored the impact of the identified nucleotide changes on protein structure using computational methods.

## Methods

### Collection and screening of blood samples

The sample collection was carried out at the Cape Coast Technical University (CCTU) clinic, in Ghana. The duration of sampling was from November 2021 to September 2022. Patients suspected of malaria were first screened for malaria infection using rapid diagnostic test (RDT) kits, Alere™ Malaria *P. falciparum* RDT (UsmRDT, Abbott, USA). Venous blood (2 mL) was collected from study participants and transferred into two separate EDTA vacutainer tubes (one for screening and DNA extraction and the other for haematological analysis). Thick and thin films were performed after RDT diagnosis. All participants and guardians of participants under age 18 were presented with an informed consent form before they were recruited into the study. Metadata including patient age, sex, and temperature were collected.

### Haematological analysis of samples

The Urit 3000Plus version 1.1 (Urit Medical Electronics, Guilin Guangxi, P.R China) was used to analyze the blood for haematological indices including WBC and haemoglobin levels. In brief, fresh venous whole blood samples were taken and transferred into a clean sample tube EDTA to keep the configuration of the WBC, and RBC. The automatic machine was set to whole blood mode and used to analyze the blood samples.

### Extraction of DNA and PCR amplification

The TIANamp genomic spin column DNA extraction kit (Tiangen, Shuangying West Road, Beijing, PR China) was used to extract DNA from the *Plasmodium*-infected blood samples following the instructions of the manufacturer. The DNA was finally eluted and stored at − 20 °C.

*Plasmodium* *falciparum-specific* infections were detected with primers targeting 18sRNA. Primers targeting the 3 coding exons of AP2-EXP2 were designed and used for PCR amplification using a uniplex assay. Primers used for the amplification are shown in Table 1. The PCR was performed in a total of 25 µL reaction containing 12.5 µL premix (Takara Bio, China), 4.0 µL DNA template 1 µL of 10 μmol L^−1^ forward primer, 1 µL of 10 μmol L^−1^ reverse primer, and 6.5 µL ddH_2_O. Cycling conditions were set at 98 °C for initial denaturation for 10 s. The second step included 35 cycles of denaturation at 98 °C for 5 s, annealing at 58.2 °C for 5 s, and elongation at 72 °C for 10 s. This was followed by a final extension temperature of 72 °C for 10 min. The amplified products were detected by electrophoresis using 1.5% agarose gel in Tris–acetate-EDTA (TAE) buffer and visualized with ethidium bromide staining.

**Table 1:** Primers for *Plasmodium falciparum* identification and AP2-EXP2 amplification

Gene name	Gene ID (Size)	Primer sequences	Target Region	Product Sizes (bp)	Reference
PFAP2-EXP2 (*Pf* apiap2)	> PF3D7_0935 (1120 bp)				
	Exon 1	> PF_0611200_467339F 5′-CACGACAGTTAAAGAAACAATGA-3′	467481	615	This study
		> PF_0611200_467907R 5′-GGGTCAAACTGTTCCTGGTG-3′	467680		
	Exon 2	> PF_0611200_467880F 5′-CAACCACACCAGGAACAGTTT-3′	467880	503	This study
		> PF_0611200_468382R 5′-CACTTCAATTTCTTTCCACCTTTTC-3′	468382		
	Exon 3	> PF_0611200_468380F 5′-TGAAAAGGTGGAAAGAAATTGAA-3′	468526	631	This study
		> PF_0611200_468959R 5′-TTGTGAAGTTATGAAGAGGTTAAAGTG-3′	468642		
*Pf* 18S rRNA (Positive Control)	M19173.1 (2040 bp)				
	* P. falciparum*	> PL1473F18 5′-TAACGAACGAGATCTTAA-3′	1473	224	This study
		> PL1679R18 5′-GTTCCTCTAAGAAGCTTT-3′	1679		

### Preparation of genomic dataset

Successfully amplified exons were sequenced using Sanger sequencing. The dataset from the field isolates was prepared in line with the preparation guidelines described elsewhere with slight modifications [23]; (1) the sequences were manually trimmed and samples with at least one quality full read (either forward or reverse) were selected. Sequences were aligned and edited according to the following steps; (2) each nucleotide base included in the consensus sequence was present in both forward and reverse sequence, and (3) no genotype was called for mixed-trace chromatograms where the variant is seen in forward and reverse reads and the peak was visible and exceed 30% of the maximum peak height. (4) variants were called wildtype if the variant signal was less than the 30% wildtype chromatogram signal. The chromatograms were examined using Seqtrace software [24]. The final consensus sequences were then aligned to a 3D7 reference genome using bioedit software to identify variants.

SNP genotypes from 801 Ghanaian isolates were obtained from the Pf6 dataset using GATK variant caller and variant call format (VCF) tools [25]. The sample collection for the Pf6 dataset ranged from 2009 to 2015. The isolates from the Pf6 dataset were collected from Cape Coast, Kintampo and Navrongo (Fig. 2b). To filter the extracted pass biallelic SNP calls with read depth (DP) ≥ 5 and allelic depth (AD) ≥ 2 were included as positive in the final dataset [26]. The final genotypes obtained from our Cape Coast field isolates were integrated with the Cape Coast samples of Pf6 MalariaGen genotypes for downstream population analysis.

### Population genetic analysis

Minor allelic frequency (MAF), polymorphic information content (PIC), and F statistics (F_ST_) for each variant were calculated using PowerMarker V3.25 software [27]. The pairwise linkage disequilibrium of the variants was estimated and visualized using the SRplot web application [28]. Tajima’s D was calculated using the sequence diversity plugin of TASSEL software version 5.2.93 [29]. Global (entire gene) and site-specific evidence of selection was detected by estimating the rate of substitutions at synonymous sites (dS) and non-synonymous sites (dN) using the SLAC tool available at the Hyhpy web-based platform [30]. All analyses were performed at an alpha value of 0.05.

### Protein modelling

Secondary structure prediction of AP2-EXP2 was done using the I-TASSER template-base algorithm web tool [31]. The secondary structure of the AP2 domain (residue 169–226) was further curated following the consensus topology of the AP2 domain, described in pioneer studies [5, 32], using Pymol software (Molecular Graphics System, v2.3. Schrodinger) [33]. Mutagenesis analysis was performed using the mutagenesis wizard function in function. The rotamers that produced the least steric clashes were selected and polar contacts were shown within 5 Å.

### Variant association analysis

Single variant association analysis was performed for the only non-synonymous mutation observed in our Cape Coast field sample—R93K—and the continuous traits using TASSEL software. The association powers for the analysis were estimated using Quanto software (version 1.2.4) [34] taking into consideration the population size, effect size and minor allelic frequency, at a 0.05% confidence interval.

## Results

### Sampling and sequencing of AP2-EXP2 coding regions

A total of 84 whole blood samples were collected of which 80 were from malaria patients and 4 were from malaria-free patients as controls for haematological analysis. Of the 80 positive samples, 72 were successfully amplified and sequenced—covering 100%, ≥ 95% and 100% nucleotide sequences of exon 1, exon 2 and exon 3 respectively. The average age, parasitaemia, temperature, white blood cell count and haemoglobin values of the successfully sequenced samples are shown in Table 2. The additional file: Dataset 1 contains individual data for the 72 successfully sequenced isolates.

**Table 2:** Demographics and haematological indices of patients whose *P. falciparum* were successfully sequenced

Parameter		Count/%	Temperature/^o^C	Haemoglobin/g/dL	WBC/ × 10^9^/L	% Parasitaemia
Gender	Female	28 (38.9%)	37.07 ± 0.60	10.7 ± 0.822	5.66 ± 1.93	18.47 ± 13.11
	Male	44 (61.1%)	37.27 ± 0.57	11.11 ± 1.93	6.41 ± 1.74	17.06 ± 11.78
Age	< 5 years	13 (18.1%)	38.02 ± 0.39	10.00 ± 0.80	6.46 ± 1.67	15.85 ± 12.38
	5–10 years	5 (6.9%)	37.35 ± 0.53	10.36 ± 1.31	6.36 ± 1.57	15.66 ± 14.56
	> 10 years	54 (75.0%)	37.08 ± 0.45	11.18 ± 1.66	6.03 ± 1.67	18.25 ± 11.82
Total		72 (100%)	37.18 ± 0.57	11.07 ± 1.66	6.21 ± 1.88	16.70 ± 12.47

Values are shown in count (percentage) and mean ± standard deviation

### Distribution and relationship analysis of variants in *Pf*AP2-EXP2

**Fig. 1:**
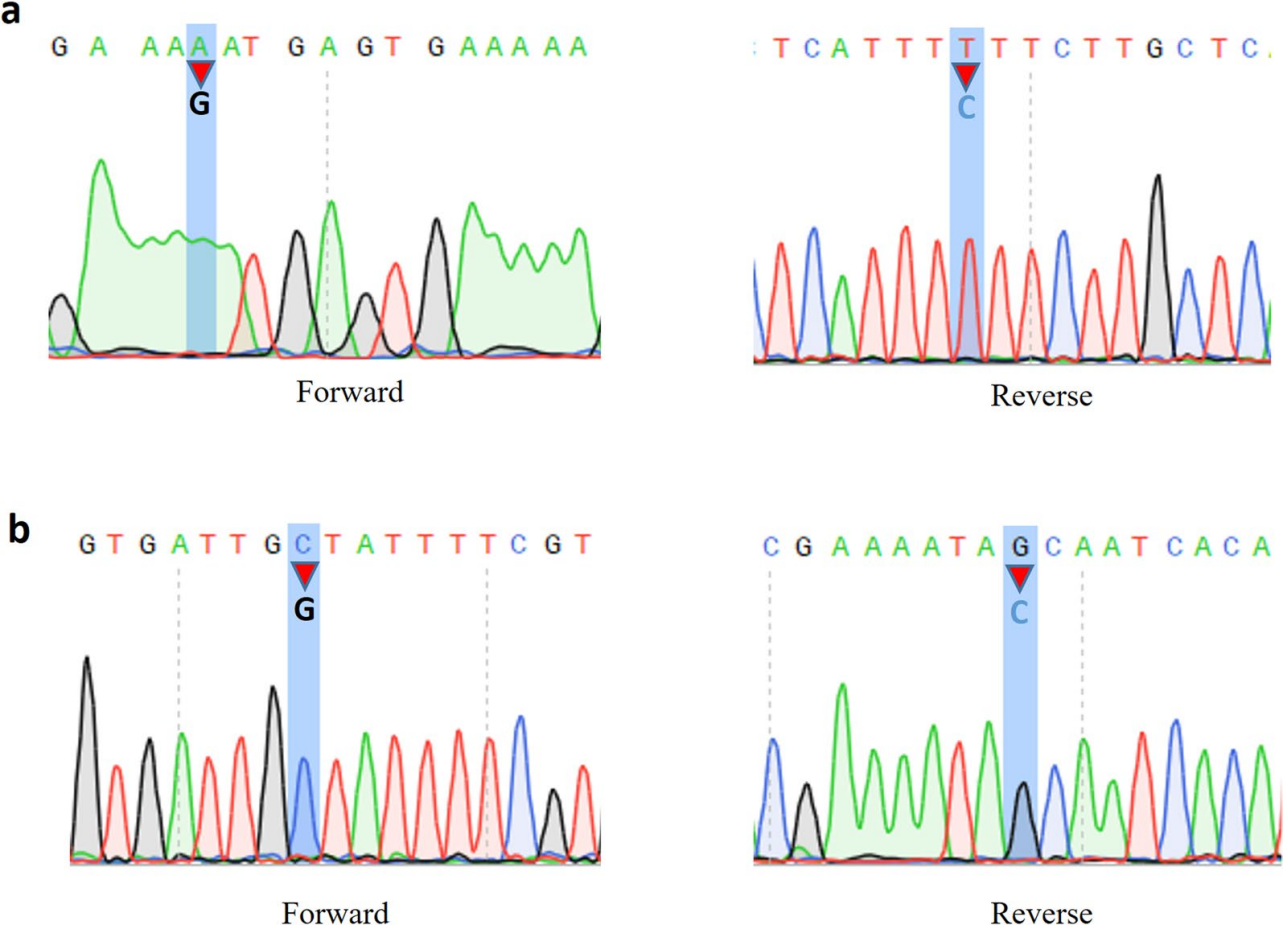
Electropherogram showing positions of variants in the field isolates. **a** Arginine-Lysine Missense SNP (mutation) at position 93 for four isolates and **b** Synonymous SNP at position 201 for three isolates

**Fig. 2:**
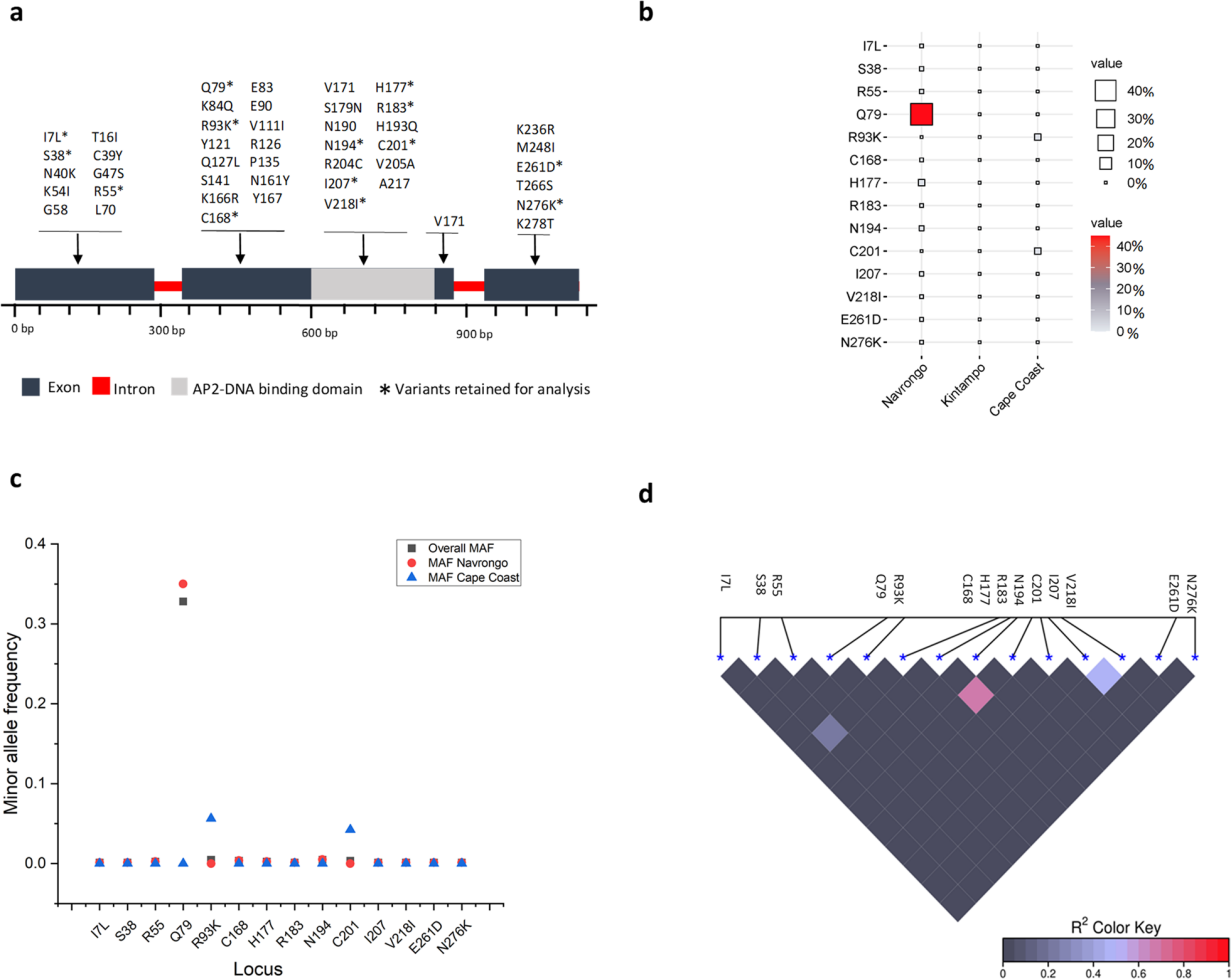
Relationship analysis of nucleotide variations in *Pf*AP2-EXP2. **a** Frequency of variants pre and post-filtration using allelic depth, * indicates variants retained after GATK filtration; **b** Percentage distribution of AP2-EXP2 of the variants among different localities. The value for each variant is shown by the size and colour of the squares; increasing percentage of the variant corresponds to increasing size of the square and the red colour **c** Visualization of Minor allelic frequency of the variants within different populations; **d** Pairwise linkage disequilibrium map for fourteen (14) loci in *Pf*AP2-EXP2 clinical isolates

**Fig. 3:**
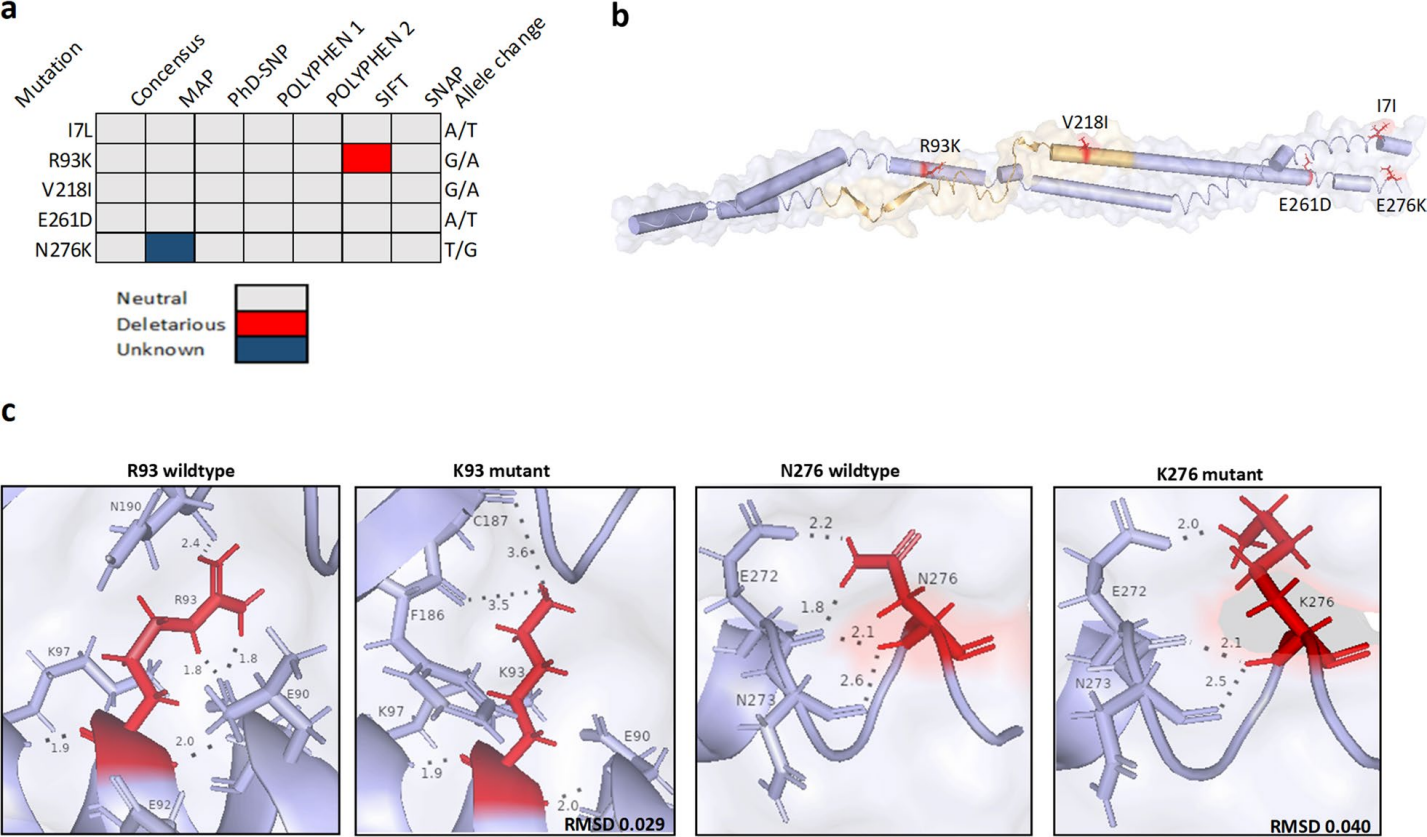
Computational prediction of the effect of missense nucleotide variations on* Pf*AP2-EXP2 protein function and structure. **a** Predictions by different web tools; consensus predictions were made based on at least four same predictions by different predicting tools; **b** Cartoon secondary structure of AP2-EXP2. Surface representation of the crystal structure of AP2-EXP2 [predicted with I-TASSER threading algorithm (C-score = − 2.44, Exp. TM-score = 0.43 ± 0.14, RMSD score 11.7 ± 4.5]. AP2 DNA-binding domain is coloured in light orange and missense variants are shown red sticks; **c** Effect of missense potentially deleterious and unknown variants on intramolecular polar contacts. Genomic variants are shown in red sticks, interaction nucleotides within 5 Å are shown in blue sticks, and polar contacts are shown in black dots

We identified two high-quality variants R93K (Phred quality score for forward sequence is 39 and reverse sequence is 62) and C201 (Phred quality score for forward sequence is 62 and reverse sequence is 62) with sharp electropherogram peaks from our 72 field samples (Fig. 1, additional file: Dataset 2). R93K and C201 were found in four [4/72 (5.5%)] and three [3/72 (4.1%)] of our Cape Coast field isolates respectively. A total of 44 variants were retrieved from the Pf6 dataset (Fig. 2a) and after filtering of variants with allele depth > 2, 12 variants were retained for downstream analysis. Notable, R93K and C201 were composites of the variants that were discarded for having allele depth ≤ 2. Because they were detected in our Cape Coast field isolates, they were included in the final variant dataset—summing up to 14 variants (Fig. 2b). Of these, nine (9) were synonymous whereas five were non-synonymous/missense variants (Fig. 2b). Except for Q79, all other identified variants were rare alleles with frequencies < 0.01 (Fig. 2c). Notably, I7L, S38, R183, I207, V218I, E261D, and N276K were singletons occurring in exactly one sample. Of the 14 nucleotide changes identified in this study, 6 (42.8%) are found within the ApiAP2 DNA binding domain, of which one, V218I was a missense mutation (Fig. 3a).

### Gesnetic diversity and natural selection

Further results showed a near-complete breakdown of linkage disequilibrium except for N194 and H177 (Fig. 2d). Gene diversity (GD) and the polymorphic information content (PIC) ranged from 0.0023 to 0.4406 and 0.0023 to 0.0337 with mean GD and PIC of 0.0379 and 0.0305 respectively. The fixation index (F_ST_) for all identified variants was 1, suggesting all variations were due to the population structure, and there is a complete lack of shared alleles between the localities (Table 3). The estimated Tajima’s D value for the entire gene was -1.6144. A global estimation of the dN/dS across the entire gene was 0.208 suggesting the presence of purifying selection acting on the gene. However, there was no site-specific evidence of selective sweep or purifying selection at a p-value of 0.05 (Table 3). Owing to the strong purifying selection observed within our clinical samples, we thought to explore dN/dS between closely related species within the same sub-genus, *Laverania.* We considered *P. falciparum* (PF3D7_0611200)*/P. reichenowi* (XP_012761928.1) and *P. gaboni* (PGSY75_0611200) genome trios retrieved from NCBI and applied dN/dS to detect selection in the *falciparum lineage*. Interestingly, we observed a comparably low dN/dS value of 0.297.

**Table 3:** Genetic variant analysis of *Pf*AP2-EXP2 in Ghanaian *Plasmodium falciparum* clinical isolates

Population	Variant	Predicted secondary structure	MAF	GD	PIC	F_ST_	dS	dN	dN-dS
Navrongo	I7L	Exposed coil	0.0011	0.0023	0.0023	1.00	0	0.3873	22.1390
Navrongo	S38	Exposed sheet	0.0011	0.0023	0.0023	1.00	1	0	− 57.1557
Navrongo	R55	Exposed helix	0.0023	0.0046	0.0046	1.00	1.0509	0	− 60.0699
Navrongo	Q79	Exposed helix	0.3280	0.4408	0.3437	1.00	1.2315	0	− 70.3876
Cape Coast	R93K	Exposed helix	0.0046	0.0091	0.0091	1.00	0	0.4988	28.5143
Navrongo	C168	Buried sheet	0.0034	0.0069	0.0068	1.00	1.5794	0	− 90.2730
Navrongo	H177	Exposed coil	0.0023	0.0046	0.0046	1.00	1.5794	0	− 90.2730
Navrongo	R183	Buried sheet	0.0011	0.0023	0.0023	1.00	0.8683	0	− 49.6312
Navrongo	N194	Exposed coil	0.0046	0.0091	0.0091	1.00	1.5794	0	− 90.2730
Cape Coast	C201	Buried sheet	0.0034	0.0069	0.0068	1.00	1.5794	0	− 90.2730
Navrongo	I207	Exposed sheet	0.0011	0.0023	0.0023	1.00	1.1357	0	− 64.9708
Navrongo	V218I	Exposed helix	0.0011	0.0023	0.0023	1.00	0	0.500	28.5778
Navrongo	E261D	Exposed helix	0.0011	0.0023	0.0023	1.00	0	0.4517	25.8180
Navrongo	N276K	Exposed helix	0.0011	0.0023	0.0023	1.00	0	0.4241	24.2398

Secondary structure prediction was made by I-TASSER algorithm. SLAC tool of Hyphy was used to estimate dN and Ds

MAF: minor allelic frequency; GD: gene diversity; PIC: polymorphic information content; F_ST:_ fixations statistics; dS: rate of substitution at synonymous sites; dN: rate of substitution at non-synonymous sites

### Effect of *Pf*AP2-EXP2 mutation of protein structure and function

Modelling of the AP2-EXP2 protein showed that its DNA-binding domain comprised three beta sheets separated by two stretches of coils and a helix towards the C-terminal end (See Fig. 3b for details of the protein modelling). The missense variations were diffused across the stretch of the protein (Fig. 3b).

We translated our *Pf*AP2-EXP2 sequence (including all missense nucleotide variants) into amino acid residues and performed multiple sequence alignments with 14 reference orthologs retrieved from NCBI. We observed that all the identified missense variants in *Pf*AP2-EXP resulted in the production of amino acids that were wildtype residues in AP2-EXP2 orthologs (Table 4). We then predicted the effect of the missense variants on protein function using PredictSNP [35]. PredictSNP combines six powerful computational predicting tools into one classifier to predict the effect of single variants on protein function. Results from PredictSNP suggested that all the identified variants were not damaging to protein function. Further, MuPro [36] prediction of the effect of the variant on thermodynamic stability suggested that 17L, R93K, V218I, E261D, and N276K reduce the thermodynamic stability of the protein with Gibbs free energy (ΔΔG) values of − 0.967, − 1.268, − 1.41, − 0.005, and − 1.466 respectively.

**Table 4:**
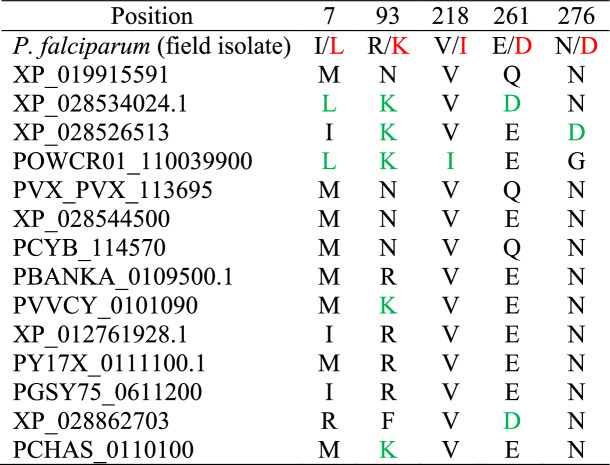
Missense mutations in AP2-EXP2 of clinical *P. falciparum* of Ghanaian origin produced new amino acid that are wildtypes in reference orthologs

Red font: Mutant residue; Green font: wildtype residue in *Plasmodium* ortholog same as the mutant residue in field isolates. Reference sequences were retrieved from NCBI and PlasmoDB databases

*P. coatneyi:* XP_019915591; *P. relictum:* XP_028534024.1; *P. gallinaceum:* XP_028526513; *P. ovale*: POWCR01_110039900; *P. vivax:* PVX_PVX_113695; *P. gondari*: XP_028544500; *P. cynomolgi:* PCYB_114570; *P. berghei:* PBANKA_0109500.1; *P. vinckei:* PVVCY_0101090; *P. reichenowi*: XP_012761928.1; *P. yoelii::* PY17X_0111100.1; *P. gaboni::* PGSY75_0611200; *P. malariae:* XP_028862703; *P. chabaudi:* PCHAS_0110100

### Mutagenesis analysis

**Fig. 4:**
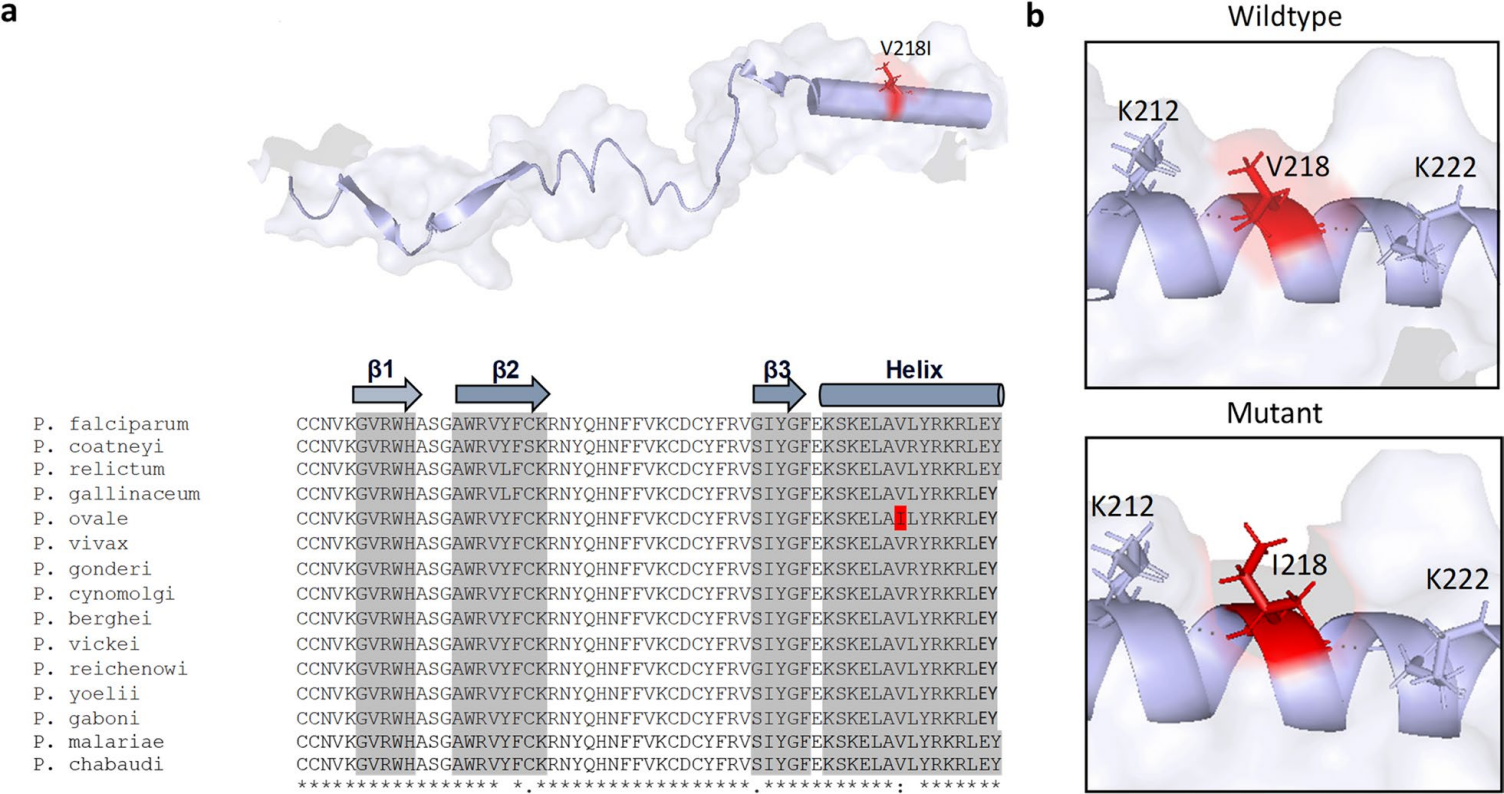
Variation in the DNA binding domain of AP2-EXP. **a** multiple sequence alignment of reference AP2-DNA orthologs using ClustalW method. The isoleucine reference allele at position 218 was highlighted in red. Secondary structure prediction was made by the I-TASSER threading algorithm and further curated according to earlier studies [5, 32]; **b** Polar contacts made by V218 and I218 were shown in black dots. (*) conserved residue; (:) conserve mutation, (.) semi-conserved mutation; () non-conservative mutation. Reference sequences were retrieved from the NCBI and PlasmoDB database. *P. falciparum*, PF3D7_0611200; *P. coatneyi,* XP_019915591; *P. relictum,* XP_028534024.1; *P. gallinaceum,* XP_028526513; *P. ovale*, POWCR01_110039900; *P. vivax,* PVX_PVX_113695; *P. gondari*, XP_028544500; *P. cynomolgi,* PCYB_114570; *P. berghei,* PBANKA_0109500.1; *P. vinckei,* PVVCY_0101090; *P. reichenowi*, XP_012761928.1; *P. yoelii,,* PY17X_0111100.1; *P. gaboni,,* PGSY75_0611200; *P. malariae,* XP_028862703; *P. chabaudi,* PCHAS_0110100

Notably, R93K was predicted by SIFT to be deleterious to protein function but all other predicting tools ranked it as a neutral variation. N276K was predicted as an unknown variation (Fig. 3a). Confirmation of this prediction using mutagenesis analysis showed that lysine substitutions at position 93 reduced the number of intramolecular polar contacts from 5 to 4 within a radius of 5 Å (Fig. 3c). Notably, wildtype interactions with E90 and N190 were broken and new bonds were formed with F186 and C187 respectively (Fig. 3c). However, amino acid residues that had interactions with the wildtype N276 were maintained in the K276 mutant and the total number of intramolecular polar bonds remained unchanged (Fig. 3c). V218I which was the only variant located within the ApiAP2 DNA-binding domain had no impact on the number of polar intramolecular bonds (Fig. 4b).

### Variant association analysis 

Association analysis under the generalized linear model (GLM) showed − log_10_ (*P*-value) values of 1.103, 1.129, 0.041 and 0.422 for temperature, haemoglobin, WBC and parasitaemia respectively, which are all below the generally adopted Manhattan plot threshold (− log_10_ (5 × 10^–8^) = 7.3) [37]. The estimated association powers for temperature, haemoglobin, WBC and parasitaemia were 72%, 39%, 5%, and 30% respectively which are also below the arbitrary 80% cutoff.

## Discussion

Recent findings have marked *Pf*AP2-EXP2 as a putative transcription factor involved in the transcriptional control of genes implicated in cellular remodelling and RBC invasion in both asexual and sexual forms, in vitro [21, 22]. Like many other transcriptional factors, the conservation of genetic sequences is important to reduce variations that could lead to the loss of normal health cell-state—[38] although this property can set them up as suitable drug targets. Here, we explored the single nucleotide variant in a known robust transcription factor, AP2-EXP2, among clinical *P. falciparum* isolates in Ghana. We found that variants in *Pf*AP2-AP2 of clinical isolates were rare variants and seemingly had no deleterious effects on protein function and structure.

It is argued that although the GATK filtering technique is designed to reduce the occurrence of sequencing errors, some residual errors remain which may end up as singleton variants [39]. This may be the case for this study or otherwise the existence of true rare alleles. The reverse instance could also occur when true positive variants are flagged as unreliable and discarded using filtering techniques. This was the case for this study as R93K and C201 were filtered out from the Pf6 dataset as variants with low read depth supporting their occurrence. We then identified these variants in our field isolates with a high-quality chromatogram on both forward and reverse sequences (Fig. 1). Against this, the number of true positives omitted due to the quality checks employed by this study on the Pf6 dataset is unknown. However, these challenges are commonplace when analysing rare alleles [39].

Rare variants are abundant and are typical footprints of natural selection [40]. As observed in this study, there was an excess of low-frequency alleles (synonymous and non-synonymous loci) in the *Pf*AP2-EXP2 genes. This observation could be explained in three folds; (1) a recent population expansion; (2) selective sweep and (3) purifying selection. The extremely low MAF, high level of singletons, and low linkage disequilibrium appear to support the first two options [40, 41]. A recent population expansion from a bottleneck effect seems a plausible explanation [42] as the *P. falciparum* genome commonly exhibits negative Tajima’s D [43]. It is worthwhile to note, that selective sweep typically has a homogenous effect on both synonymous and non-synonymous variants producing excess rare variants across a given locus [42], but this was not the case for Q79 (which had MAF of approximately 33% as opposed to the < 1% MAF for all the other variants). Inferably, Q79 could be a hitchhiker that resided on a selected haplotype during a selective sweep. However, the restrained genomic location considered by this study could not allow the detection of the distant selected beneficial allele if indeed the gene was under selective sweep. Nonetheless, a more plausible explanation would be that, Q79 is likely a neutrally evolving synonymous variant that suffered little to no effect during a purifying selection. Evidently, the global within- and between species dN/dS observed in this study were far below one (1), which is consistent with the expectation that the essentiality of AP2-EXP2 predisposes it to purifying selection to maintain protein structure and function. Thus *Pf*AP2-EXP2 in the clinical Ghanaian isolates is reminiscent of a gene that has undergone a recent population expansion and experiencing purifying selection. In concordance with our findings, *Pf*AP2-EXP2 from different West African countries did not show any signal of selective sweep [16–18] despite the widespread signals within its vicinity. It remains to be demonstrated if AP2-EXP2 from these regions exhibit a similar pattern of purifying selection as observed in this study.

It is believed that protein-coding genes with similar protein structure and/or function tend to have a similar evolutionary fingerprint [44], and could be revealed by dN/dS estimates. In concert with this posit, we observed that *Pf*AP2-EXP2 of Ghanaian origin is subjected to equally strong purifying selection, of comparable magnitudes, as observed between the closely related species. The observed long-term conservation of strong purifying selection could be critical to maintaining the essential function of AP2-EXP2 within the *Laverania* subgenus.

As widely accepted, purifying selection removes strong deleterious variants from a given evolving population [45]. To verify the inference made in this study so far, we combined a simple between-species multiple sequence alignment with bioinformatics estimates. A comparative sequence alignment of the mutants, interestingly revealed that all the identified missense mutations resulted in the production of mutant residues that are reference alleles in AP2-EXP2 orthologs (Table 4). This suggests they are likely non-deleterious missense mutants that pose no deleterious effect on the protein structure and function just like the synonymous mutants [46]. Contrastingly, R93K and N276K were predicted to be deleterious and unknown by one of the predicting tools respectively (Fig. 3). The tentative deleterious effect of R93K could explained by the fact that the guanidine group in Arginine allows for interactions in three possible directions forming a higher number of polar contacts and salt bridges [47]. However, the functional group of lysine allows interaction in just one direction, generating fewer polar bonds with surrounding amino acids [47, 48]. Other thoughts explain that substituting arginine for lysine is preferred owing to the similarities in their biochemical properties. To validate these predictions, we estimated the degree of structural destabilization induced by the R93K and N276K by mutagenesis analysis and used root mean square deviation (RMSD) as an index to measure the degree of deviation from the native 3D structure. It has been estimated that RMSD above 0.4 Å signifies a substantial local change to 3D structures whereas RMSD below 0.2 Å denotes a negligible effect on the conformation of the native structure [49]. In comparison with these thresholds, neither R93K nor N27K was destabilizing enough to cause a discernible effect on the three-dimensional structure of AP2-EXP2 suggesting a strong structural conservation of AP2-EXP2 among clinically circulating *Plasmodium falciparum* isolates.

Recently, a variant in the DNA-binding domain of an ApiAP2 member, PBANKA_011210, has been experimentally shown to be associated with host phenotypes; immune response and the development of cerebral malaria in rats [14, 15]. Whether variants in other ApiAP2 members and their syntenic orthologs show association with host phenotypes is an outstanding question. In this present observational study, we observed no significant association between the identified variant and host factors of humans. This may reflect a true low effect size—a measure of the difference in host phenotype between a variant and a wildtype. Another factor that could have influenced the observed association is sample size. Against this, there will be the need to perform a variant association analysis with a larger sample size or perform experimental analysis to verify these findings in future studies. This study sets the premise for exploring the functional and structural conservation of the ApiAP2 gene family in clinical isolates which could inform future research directions.

## Conclusions

*Pf*AP2-EXP2 showed low nucleotide diversity among clinical isolates from Ghana consistent with purifying selection acting on this essential gene. This is contrary to what is observed for genes close to its vicinity among West African *P. falciparum* isolates. The variants were not associated with host factors determined from our clinical samples. Further study is needed to validate the essentiality of this gene in other *Plasmodium* species, including other human-infective species that are important for assigning AP2-EXP2 as an antimalarial drug target.

## Supplementary Information

Additional file 1: Dataset 1. Demographic and haematological data of study participants recruited in our field study. Dataset 2. Genotypic data of successfully sequenced *Pf*AP2-EXP2 gene of 72 field samples collected in Cape Coast.

## Data Availability

No datasets were generated or analysed during the current study.
